# Correlates of bullying perpetration among Lebanese adolescents: a national study

**DOI:** 10.1186/s12887-021-02678-0

**Published:** 2021-04-28

**Authors:** Emmanuelle Awad, Chadia Haddad, Hala Sacre, Rabih Hallit, Michel Soufia, Pascale Salameh, Sahar Obeid, Souheil Hallit

**Affiliations:** 1 Faculty of Social Sciences, Psychology Department, Lund University, Lund, Sweden; 2 Research and Psychology Departments, Psychiatric Hospital of the Cross, Jal Eddib, Lebanon; 3 INSERM, Univ. Limoges, CH Esquirol, IRD, U1094 Tropical Neuroepidemiology, Institute of Epidemiology and Tropical Neurology, GEIST, Limoges, France; 4 INSPECT-LB: National Institute of Public Health, Clinical Epidemiology and Toxicology, Beirut, Lebanon; 5 Faculty of Medicine and Medical Sciences, Holy Spirit University of Kaslik (USEK), Jounieh, Lebanon; 6 University of Nicosia Medical School, Nicosia, Cyprus; 7 Faculty of Pharmacy, Lebanese University, Hadat, Lebanon; 8 Faculty of Arts and Sciences, Holy Spirit University of Kaslik (USEK), Jounieh, Lebanon

**Keywords:** Adolescents, Schools, Bullying, Correlates, Lebanon

## Abstract

**Background:**

Presently, 1 in 4 Lebanese adolescents is involved in bullying, with 12% being perpetrators. In Lebanon, around 90% of bullying incidents occur in schools. Given the lack of studies tackling bullying perpetration in Lebanon, this study aims to identify and target risk factors of bullying perpetration among Lebanese adolescents, which would serve future prevention and intervention programs.

**Methods:**

This cross-sectional study took place between January and May 2019 in a proportionate random sample of schools from all Lebanese districts. A total of 1810 (90.5%) out of 2000 students accepted to participate.

**Results:**

Results showed that 831 (49.1%, CI: 0.46–0.51) participants had bullied other people. A stepwise linear regression, using as the dependent variable the bullying perpetration score, showed that higher psychological (β = 0.12; 95% CI 0.083–0.176), sexual (β = 0.26; 95% CI 0.128–0.411), neglect (β = 0.08; 95% CI 0.051–0.120), physical abuse (β = 0.13; 95% CI 0.036–0.235), higher internet addiction (β = 0.07; 95% CI 0.057–0.097), higher social fear (β = 0.10; 95% CI 0.075–0.140), and having separated parents (β = 1.60; 95% CI 0.561–2.650) were significantly associated with more bullying perpetration. Higher social avoidance (β = − 0.03; 95% CI -0.062- -0.003) was significantly associated with less bullying perpetration.

**Conclusion:**

The results revealed that bullying perpetration is significantly associated with parental status, child abuse, internet addiction, and social fear. Educational and relevant governmental institutions could use our findings to develop and implement efficient bullying prevention and intervention programs for all involved parties.

## Background

Bullying perpetration, a dangerous phenomenon that has become the focus of global research, is an intentional, aggressive pattern of behavior repeatedly and consistently carried out over some time by a single individual or group, often associated with a leader. It is employed as an expression of real or perceived power imbalance and dominance [1]. Several types of bullying perpetration exist, e.g., verbal, physical, social, relational, and cyber, the latter being the most common. Verbal bullying could be name-calling, threatening, mocking, and making derogatory remarks. Physical bullying includes hitting, shoving, destroying the victim’s possessions, tripping, and spitting. Social or relational can take the form of spreading false rumors, exclusion, or any actions meant to hurt the victim’s reputation or social standing. Cyberbullying is humiliating, intimidating, and inflicting harm upon someone through the Internet [2]. A recent study conducted in India showed that 5.2% of adolescents aged between 12 and 15 engage in bullying perpetration [3]. In 2018, the Middle East and North Africa region was listed among the countries most vulnerable to bullying among adolescents aged 11 to 15 [4]. Bullying perpetration had the lowest percentages in Northern Europe while Baltic countries showed higher numbers among European countries, with prevalence ranging from about 8 to 45% overall [5].

### Factors related to bullying perpetration

Various theories sought to study the contributing variables behind bullying perpetration, confirming that catalysts from multiple sources are involved [6]. Aggressive behavior towards others results from biological, psychological, and social vulnerabilities such as genetic predispositions, personality traits, and childhood events [7]. However, over the past few years, evidence suggests a bidirectional relation between psychological and social factors contributing to bullying perpetration, regardless of biological predispositions [8].

### Individual factors related to bullying perpetration

Studies across countries confirm that males are consistently more likely to engage in all forms of bullying perpetration [9]. Men who exhibit traditional masculine stereotypes were more likely to engage in bullying perpetration [10].

A previous study found a positive correlation between the consumption of psychoactive substances, specifically cigarettes and alcohol, and maladaptive behaviors such as bullying perpetration [11]. A higher Body Mass Index (BMI) was also positively linked to bullying perpetration [12], with a greater prevalence of obese or overweight students among bullies [13].

### Psychological factors related to bullying perpetration

Psychological factors also offer insight into bullying perpetration. Depressive symptoms and suicidal ideation can exist in both bullied and perpetrators [14]. Perpetrators of traditional bullying were more likely to have higher levels of anxiety, particularly social [15]. Moreover, adolescents who reported to be internet addicts were more at risk of becoming or being currently bullied [16].

### Social factors related to bullying perpetration

Dysfunctional family dynamics correlated with bullying perpetration [17]. Furthermore, childhood maltreatment, such as harsh punitive parental styles, was associated with bullying perpetration during adolescence [18]. Parenting that consistently included strictness and punishment was positively correlated with bullying behaviors [19]. Additionally, adolescents living in crowded homes, relating to the number of bedrooms, engage in more antisocial behavior, including bullying perpetration [20].

### Rationale and purpose of the study

Presently, 1 in 4 Lebanese adolescents is involved in bullying, with 12% being perpetrators [21]. In Lebanon, around 90% of bullying incidents occur in schools [22]. The current lack of studies assessing individual and contextual variables associated with bullying perpetration in Lebanon is alarming, given the high prevalence of local incidents. Therefore, this study aims to identify and target sociodemographic, psychological, and contextual factors of bullying perpetration among Lebanese adolescents, which would serve future prevention and intervention programs.

## Methods

### Participants

This study was started on January 2019 and lasted for 5 months. It is a cross-sectional design based on a proportionate sample drawn out of all Lebanese Mohafazat/districts (Beirut, Mount Lebanon, North, South, and Beqaa). Of the 18 non-public schools approached (4 in Beirut, 2 in the South, 6 in Mount Lebanon, 2 in the North, and 2 in Beqaa), 16 accepted to participate, and two declined. One thousand eight hundred ten questionnaires out of 2000 distributed were collected. All students between 14 and 17 were eligible. Previous description of the methodology in this paper can be found elsewhere [23–27].

### Sample size calculation

A minimum of 652 persons was needed according to the G-power software, based on an effect size f2 = 2%, an α error of 5%, a power of 95%, and 12 variables to be entered in the multivariable analysis.

### Questionnaire

The questionnaire distributed was in Arabic, and necessitated around an hour to complete. Data were collected anonymously during school time, in the classrooms, to eliminate parental influence.

The first section of the questionnaire consisted of sociodemographic questions. Self-reported values (kg/m^2^) were used to compute the Body Mass Index (BMI). The household crowding index (HCI) is the ratio of the number of people residing in the apartment over the number of rooms available, excluding the kitchen and bathrooms. The second section comprised of the different scales used:

#### The Illinois bully scale

Validated in Lebanon [28], this scale measures bullying perpetration and victimization. Higher reported scores indicate higher bullying perpetration (in this study, Cronbach’s α = 0.975) [29].

#### The Liebowitz social anxiety scale (LSAS)

This tool is constituted by 2 subsections made of 24 items: 13 relating to performance anxiety (fear) and 11 concerning social settings (avoidance). Fear and avoidance are proportionally related to the score (in this study, Cronbach’s α total score = 0.969, Cronbach’s α fear = 0.952, Cronbach’s α avoidance = 0.951) [30].

#### The internet addiction test (IAT)

This test is validated in the country and is available in the Arabic language and consists of 20 items; higher scores indicate a higher internet addiction (in this study, Cronbach’s α = 0.925) [27, 31].

#### The adolescent depression rating scale (ADRS)

Ten dichotomous questions (yes/no) constitute this scale that screens for depression among adolescents. Higher scores indicate higher levels of depression (in this study, Cronbach’s α = 0.940) [32].

#### Child abuse self-report scale (CASRS)

This scale is made of 38 questions and measures four types of child abuse: physical (8), psychological/emotional (14), sexual (5) and neglect (11). Higher scores indicate higher child abuse (in this study, Cronbach’s α physical = 0.966, Cronbach’s α psychological = 0.973, Cronbach’s α sexual = 0.954, Cronbach’s α neglect = 0.971) [33].

### Translation procedure

All the scales, except the IAT, were translated using the forward-backward method. Two study-independent translators were involved, the first translated from English into Arabic, and the second performed the back-translation. Differences between the original and the English translated versions were resolved by agreement.

### Statistical analysis

Data were analyzed using SPSS software version 25. Cronbach’s alpha measured reliability, Pearson’s coefficient calculated the correlation between continuous variables, and the Student t-test compared the means between two groups. We used Stepwise linear regression choosing as the dependent variable the total bullying score. The Bonferroni correction was applied to calculate the statistical significance of bivariate analysis [34]. Twelve hypotheses/variables were checked for possible association with the bullying perpetration variable, with the desired error *a* = 0.05; therefore, the Bonferroni correction would test each hypothesis at a *p* = 0.05/12 = 0.004. Independent variables that showed a *p* < 0.004 in the bivariate analysis were entered in the final model,. In the multivariable analysis significance was defined as *p* < 0.05.

## Results

The mean age was 15.42 ± 1.14 years, with 53.3% of females. Table 1 shows other sociodemographic parameters of the participants.

**Table 1:** Sociodemographic characteristics of the sample population

	**Frequency (%)**
**Gender**	
Male	844 (46.7%)
Female	963 (53.3%)
**Parents status**	
Living together	1581(88.1%)
Separate	213 (11.9%)
**Smoking status**	
Yes	468 (25.9%)
No	1342 (74.1%)
	**Mean ± SD**
**Age (years)**	15.42 ± 1.14
**Body Mass Index (kg/m2)**	21.95 ± 4.21
**Household crowding index**	1.01 ± 0.64

### Scales description

The mean bullying score was 8.23 ± 8.03, and the median was 6. Since there were no cutoff points for this scale, the median was used as the cutoff point; 831 (49.1%, CI: 0.46–0.51) students had bullied other people (Table 2).

**Table 2:** Description of the scales used

	Mean ± SD	Median	Minimum	Maximum	Range
**Bullying score**	8.23 ± 8.03	6.00	0	27	0–36
**Depression score**	4.64 ± 2.10	4.68	0	10	0–10
**Child psychological abuse scale**	10.63 ± 11.49	7.00	0	42	0–42
**Child abuse neglect scale**	13.91 ± 10.70	12.00	0	33	0–33
**Child abuse physical scale**	5.74 ± 6.70	2.00	0	24	0–24
**Child abuse sexual scale**	2.79 ± 4.01	0	0	15	0–15
**Liebowitz fear score**	26.17 ± 16.15	27.00	0	72	0–72
**Liebowitz avoidance score**	30.95 ± 16.94	33.00	0	72	0–72
**Internet addiction scale**	39.42 ± 18.08	40.00	0	100	0–100

### Bivariate analysis

Students with separated parents scored higher on the bullying scale (12.55 vs. 7.63, *p* < 0.001). A similar result was seen in smokers compared to non-smokers (10.56 vs. 7.44, *p* < 0.001). Higher bullying scores were significantly associated with fear, avoidance, addiction to the internet, depression, neglect, physical and sexual abuse, whereas lower bullying scores were correlated to higher HCI (Table 3).

**Table 3:** Bivariate analysis taking the bullying scale as the dependent variable

	**Bullying scale**	***P*** **-value**
	**Mean ± SD**	
**Parents status**		
Living together	7.63 ± 7.90	**< 0.001**
Separated	12.55 ± 7.53	
**Smoking status**		
Yes	10.56 ± 7.49	**< 0.001**
No	7.44 ± 8.06	
	**Correlation coefficient**	***P*** **-value**
Age	−0.045	0.067
Liebowitz- fear score	0.375	**< 0.001**
Liebowitz- avoidance score	0.226	**< 0.001**
Internet addiction	0.266	**< 0.001**
House crowding index	−0.068	**0.005**
Depression score	0.146	**< 0.001**
Psychological abuse scale	0.424	**< 0.001**
Child abuse neglect scale	0.259	**< 0.001**
Child abuse physical scale	0.387	**< 0.001**
Child abuse sexual scale	0.350	**< 0.001**

### Multivariable analysis

A stepwise linear regression, using as the dependent variable the bullying perpetration score, showed that higher psychological (β = 0.12; 95% CI 0.083–0.176), sexual (β = 0.26; 95% CI 0.128–0.411), neglect (β = 0.08; 95% CI 0.051–0.120), physical abuse (β = 0.13; 95% CI 0.036–0.235), higher internet addiction (β = 0.07; 95% CI 0.057–0.097), higher social fear (β = 0.10; 95% CI 0.075–0.140), and having separated parents (β = 1.60; 95% CI 0.561–2.650) were significantly associated with more bullying perpetration (Table 4). Results were considered adjusted over all variables that showed a non-significant association in the bivariate analysis.

**Table 4:** Linear regression taking the bullying perpetration score as the dependent variable

	Unstandardized Beta	Standardized Beta	***p***-value	Confidence Interval	
				Lower	Upper
Child psychological abuse	0.129	0.175	< 0.001	0.083	0.176
Child sexual abuse	0.269	0.125	< 0.001	0.128	0.411
Liebowitz fear score	0.107	0.218	< 0.001	0.075	0.140
Internet addiction	0.077	0.174	< 0.001	0.057	0.097
Child abuse neglect scale	0.086	0.106	< 0.001	0.051	0.120
Parents status (separated vs together^a^)	1.605	0.068	0.003	0.561	2.650
Child physical abuse	0.135	0.105	0.008	0.036	0.235
Liebowitz avoidance score	−0.032	−0.067	0.033	−0.062	−0.003

^a^Reference group; Variables entered in the model: parents’ status, internet addiction, Liebowitz fear score, Liebowitz avoidance score, depression, psychological abuse scale, child abuse neglect scale, child abuse physical scale and child abuse sexual scale; Adjusted R^2^=0.336.

## Discussion

Multiple social and psychological factors are associated with bullying perpetration. Our results showed that higher bullying perpetration scores were positively associated with having separated parents, all types of child abuse, higher social fear, and internet addiction.

A previous Lebanese study had shown that 19.8% of Lebanese students in Beirut engaged in bullying perpetration at the time of investigation [35]. Our nationally representative study revealed that 41.9% of school-attending adolescents currently engage in bullying perpetration, demonstrating a higher prevalence among the Lebanese population. In contrast with previous findings, the percentage of bullying perpetration in Lebanon is significantly greater than that in Italy (20.9%) [36], China (16.3%) [37], and the USA (19.4%) [38]. The percentage among Lebanese students should be treated with caution since the bullying scale has no cut-off point.

### Bullying perpetration and child abuse

Our results showed that all types of child abuse were associated with higher bullying perpetration scores. A comparative study showed that physical abuse is positively associated with bullying perpetration, suggesting that victims of physical maltreatment are more likely to cope with the trauma by engaging in bullying behaviors at a later stage [39]. Canadian longitudinal studies demonstrated that children who suffered from physical and/or verbal abuse are at a higher risk of engaging in aggressive behavior such as bullying perpetration [40]. Child researchers also found that emotional and physical neglect occurring during childhood are likely to produce violent tendencies in adolescence. Fear and avoidance are also associated with past abuse; adolescents who suffered from child abuse are more likely to have difficulties recognizing and regulating internalizing problems, which may later translate into externalizing behavior [41].

Several theories attempted to explain bullying perpetration from a psychosocial perspective. According to the diathesis-stress model, maladaptive behavior occurs as an externalizing consequence of collaboration between internal or psychological and social stressors [42]. Parental conflict, hostile home environment, and abuse are considered diatheses or stressors, combined with cognitive, emotional, or biological vulnerabilities, increase the possibility of bullying perpetration among adolescents [6]. Similarly, the general strain theory suggests that individuals experiencing environmental pressures are more likely to engage in pathological behavior [43]. The strains can include low parental involvement or neglect and physical or psychological abuse from main characters in the child or adolescent’s life, which then lead to bullying others and potential delinquency [44]. Referring to the social learning theory, aggressive behavior towards others, including bullying perpetration, can be learned through observation (by witnessing domestic violence or parental abuse, and subsequently modeling or imitating) if the behavior is perceived as rewarding [45, 46]. Individuals who have experienced abuse or parental aggression are more likely to engage in bullying perpetration during adolescence [47]. In essence, the core belief among psychosocial theories is that bullying perpetration results from an interaction between psychological and ecological risk factors.

### Bullying perpetration and social fear

Our results showed a significant relationship between bullying perpetration and social fear. Verbal and relational bullying perpetrators were more likely to exhibit higher social fear levels than non-perpetrators or physical bullies [48]. Furthermore, a longitudinal study showed that anxiety symptoms have a cascading effect on both being bullied and bullying others [49]. Previous research reviews suggest the possibility of internalizing problems, specifically depression and social anxiety, serving as mediating factors between ecological variables such as divorce and child abuse, and bullying as an externalizing behavior [50]. Failure to develop adequate social communication skills due to social fear might manifest in maladaptive behaviors such as aggression, defensiveness, and confrontation, which are actions associated with bullying perpetration [15]. Lebanon fosters a collectivist society, which focuses on the group instead of the individual, which could discourage the emotional expression or acquisition of appropriate communication skills to bully others [51].

### Bullying perpetration and internet addiction

Internet and mobile phone addiction had a direct effect on bullying perpetration among younger South Korean adolescents [52]. Additionally, students who had Problematic Internet Use (PIU) and those who spent more time online than others were more likely to report bullying others [16, 53]. Overall, the association between bullying perpetration and internet addiction in our sample showed consistency with global studies. Furthermore, compulsive use of the internet correlated with cyberbullying perpetration [54]. Previous studies had found that PIU is a predictor of cyberbullying: the more time spent using the Internet, the higher the probability of cyberbullying perpetration [53]. The positive relationship between these two variables could result from an interaction between individual and contextual factors. Adolescents presenting negative emotional symptoms such as anxiety and depression and experiencing low parental involvement are more likely to be internet addicts and bullying perpetrators [55].

### Bullying perpetration and parents’ status

The most influential contextual variable to consider when investigating bullying perpetration is the adolescent’s environment. The outcome relating to the parents’ marital status contrasts with a previous Lebanese study where no significant relation was found between bullying perpetration and having married, separated, or deceased parents [56]. However, previous research showed that lower parental involvement is associated with a higher probability of bullying perpetration among adolescents [57]. Having parents who are separated or not living in the same house is considered a risk factor for adolescents in the Netherlands [58]. In Canada, families of divorced parents have a higher prevalence of bullies [59]. A longitudinal study conducted in South Korea showed that adolescents living with a single parent are more likely to be perpetrators of traditional bullying [14]. Adverse life events, especially childhood experiences that involve family, can be predictors of adolescent bullies. Bullying perpetration could be a means of externalizing anger, anxiety, or depression resulting from previous negative events such as divorce or parental separation [18]. Several longitudinal studies showed that adolescents living in homes characterized by constant conflicts are more likely to engage in problematic and violent behaviors, including bullying others [60]. The Social Learning Theory (SLT) suggests that witnessing aggressive behavior between parents might model this action to the child, which learns and implements it later during adolescence. Bullying behaviors can also be learned and reinforced by peers or abusive caregivers other than the child’s parents [61].

### Clinical implications

Bullying affects perpetrators, victims, and bystanders; its long-term effects are detrimental. Based on our results and aided by the validated Illinois Bully scale, officials at educational institutions and mental health experts can develop and implement evidence-based anti-bullying programs targeting the risk factors or associated variables to prevent further bullying incidents and effectively handle the repercussions.

Given that our findings involve both social and psychological constituents, measures should take a socio-ecological approach. The social-ecological theory has been mainly useful in conceptualizing traditional (face-to-face, verbal, and relational) forms of bullying [47]. This approach acknowledges that health risks are not direct consequences of distinct actions/conducts. Instead, they appear to be the result of multifaceted interactions among individuals and the environments they live in [5]. Thus, to convincingly address cyberbullying actions, an environmental framework should tackle ecological, cognitive, and psychosocial risks and protective factors at the individual, family, peer, online, and community levels. Therefore, interventions should not be exclusive to involved individuals but also include teacher training and frequent parental meetings. Active cooperation between schools, parents or caregivers, and the adolescents involved in bullying is imperative for a successful outcome.

### Limitations and strengths

Our study has some limitations. First, a selection bias is present due to the refusal rate and because it did not enroll any public schools. Second, data were obtained through a self-reported questionnaire, which may increase bias. Third, some of the scales used are not validated in the Lebanese population but were translated using the forward-backward translation method. A residual confounding bias is also possible since not all factors associated with bullying perpetration were considered in this study.

Our study presents some strengths as well. To our knowledge, it is the first to examine the relationship between bullying perpetration and several social and psychological variables in Lebanon. Additionally, results can be generalized to the population of adolescents attending local private schools only, as the sample was representative, selected proportionally, and randomly.

## Conclusion

This study has public health value and could shed light on social and psychological factors related to bullying perpetration among adolescents. The results revealed that bullying perpetration is significantly associated with parental status, child abuse, internet addiction, and social fear. Educational and relevant governmental institutions could use our findings to develop and implement efficient bullying prevention and intervention programs for all involved parties.

## Data Availability

The authors do not have the right to share any data information as per the ethics committee policy.

## Abbreviations

LSAS: Liebowitz Social Anxiety Scale
IAT: Internet Addiction Test
ADRS: Adolescent depression rating scale
CAS: Child abuse self-report scale
KMO: Kaiser-Meyer-Olkin
RMSEA: Root Mean Square Error of Approximation
GFI: Goodness of Fit Index
AGFI: Adjusted Goodness of Fit Index
